# Validity and practical utility of accelerometry for the measurement of in-hand physical activity in horses

**DOI:** 10.1186/s12917-015-0550-2

**Published:** 2015-09-11

**Authors:** R. Morrison, D. G. M. Sutton, C. Ramsoy, N. Hunter-Blair, J. Carnwath, E. Horsfield, P. S. Yam

**Affiliations:** School of Veterinary Medicine, College of Medical, Veterinary and Life Sciences, University of Glasgow, 464 Bearsden Road, Glasgow, G61 1QH Scotland

**Keywords:** Horse, Physical activity, Accelerometry, Obesity, Validation

## Abstract

**Background:**

Accelerometers are valid, practical and reliable tools for the measurement of habitual physical activity (PA). Quantification of PA in horses is desirable for use in research and clinical settings. The objective of this study was to evaluate a triaxial accelerometer for objective measurement of PA in the horse by assessment of their practical utility and validity.

Horses were recruited to establish both the optimal site of accelerometer attachment and questionnaire designed to explore owner acceptance. Validity and cut-off values were obtained by assessing PA at various gaits. Validation study- 20 horses wore the accelerometer while being filmed for 10 min each of rest, walking and trotting and 5 mins of canter work. Practical utility study- five horses wore accelerometers on polls and withers for 18 h; compliance and relative data losses were quantified.

**Results:**

Accelerometry output differed significantly between the four PA levels (*P* < 0•001) for both wither and poll placement. For withers placement, ROC analyses found optimal sensitivity and specificity at a cut-off of <47 counts per minute (cpm) for rest (sensitivity 99.5 %, specificity 100 %), 967–2424 cpm for trotting (sensitivity 96.7 %, specificity 100 %) and ≥2425 cpm for cantering (sensitivity 96.0 %, specificity 97.0 %). Attachment at the poll resulted in optimal sensitivity and specificity at a cut-off of <707 counts per minute (cpm) for rest (sensitivity 97.5 %, specificity 99.6 %), 1546–2609 cpm for trotting (sensitivity 90.33 %, specificity 79.25 %) and ≥2610 cpm for cantering (sensitivity 100 %, specificity 100 %) In terms of practical utility, accelerometry was well tolerated and owner acceptance high.

**Conclusion:**

Accelerometry data correlated well with varying levels of in-hand equine activity. The use of accelerometers is a valid method for objective measurement of controlled PA in the horse.

## Background

Accelerometers are small, non-invasive, portable devices that can be used to quantify the intensity, duration and frequency of physical activity (PA). These devices have been used extensively over the past decade in humans, both adults and children, and more recently in animals including dogs [1–4] cats [5] and cows [6] to measure the amount and frequency of movement. However, few studies have reported using accelerometers for the measurement of PA in horses [7].

In humans, accelerometers are typically worn on the hip, close to the body’s centre of mass, and can accurately record PA without incurring recall bias from the subject [8]. Accelerometry is now regarded as a reliable mechanism with which to collect information on free-living habitual PA [9–11]. It has facilitated a number of studies in which PA levels are of interest such as in obesity where accelerometry has been used to improve the knowledge of its aetiology, prevention and treatment [12–17].

In veterinary medicine, accelerometers have been shown to be valid, practical and reliable for the measurement of habitual PA in dogs [2] and subsequently the technique has been applied for objective evaluation of PA in dogs during a weight loss programme [18]. As the devices are portable, lightweight and non-invasive they could potentially be used in horses for measurement of PA. As in other species, equine obesity has a relatively high prevalence and estimates in UK and Scotland range from 21 to 45 % [19, 20] and predisposes to important conditions such as laminitis and equine metabolic syndrome [21]. The ability to quantitatively and objectively monitor the PA levels of a horse using accelerometers is highly desirable, however as in humans, requires evaluation and validation prior to it being used for research into PA in relation to obesity or many of the other potential applications. The measurement of PA in horses has been validated previously using accelerometer multidirectional accelerometer^1^ [22] and more recently with a pedometer^2^ [7]. However, as yet there are no published studies that describe using one of the most commonly used triaxial accelerometers^3^ in human PA research to measure PA in horses.

The purpose of this investigation was to evaluate a triaxial accelerometer for the objective measurement of PA in the horse by assessment of its practical utility, and validity during controlled PA. Firstly, it was important to establish that the accelerometer was practical to use in the turned out horse and that it is well tolerated by horses and acceptable to owners. For this, the optimal site of attachment of the accelerometer needed to be determined. Secondly, in human and animal studies, accelerometry is usually validated against either directly observed movement or against energy expenditure, both methods being regarded widely as ‘gold standards’ for the validation of movement [2, 9–11]. The objective of the validation was to test that accelerometry output increased significantly as directly observed intensity of in-hand equine PA increased. Support for this hypothesis would thus support the validity of accelerometery for measuring controlled PA in the horse.

## Methods

### Accelerometer settings

Two studies were undertaken–practical utility, and validation. The accelerometer used during these studies is a small, water-resistant, portable device (46 × 33 × 15 mm, weighing 19 g) that records acceleration along three axes (defined as X, Y, Z by the manufaturer). Powered by a lithium ion polymer battery, it is capable of recording accelerations in the range ±6 g (gravitational acceleration) for up to 30 days. The GT3-X+ can record data in sampling rates between 30 and 100 Hz, as specified by the user. In this study the accelerometer was set to sample date at 30 Hz. The raw data is then band pass filtered at a bandwidth of 0.25 to 2.5 Hz intended to eliminate high frequency artefact vibrations, and converted to an activity count using a proprietary algorithm. The recorded activity counts are then summed over a period of time specified by the user, known as an epoch. In these investigations the accelerometer recorded the PA of each horse in 15 s epochs, but these were then summarised over 1 min periods to give a mean accelerometer count per minute. Activity data recorded by the accelerometer can be downloaded using the manufacturers accompanying software^4^ which outputs the activity countfor each individual axis and also a calculated vector magnitude of the three individual axes combined.

### Practical utility study

Initially the most suitable site of attachment on the horse and practical utility of the accelerometer were investigated in a convenience sample of five horses. Evaluation of the practical utility of each potential site was considered to include ease of attachment, longevity of attachment and data loss/absence. Cable ties were used to attach the accelerometer to the head collar at the poll of the horse. For the withers, each horse either wore a nylon stretch bib usually worn to protect the shoulders, chest and withers from rug rub, with accelerometers attached via hook and loop tape or attachment of an accelerometer to a surcingle 10 cm below the withers. Duct tape was used to attach accelerometers to the sternum and the sacrum.

Five accelerometers at the aforementioned sites, recording PA in 15 s epochs, were worn simultaneously by each horse for 2 h over different levels of PA. Any problems experienced with the accelerometers over the study period were noted by one of the researchers.

Practical utility of attachment of the accelerometers to the poll and withers (attachment to a head collar and surcingle respectively) was further investigated for 18 consecutive hours in five horses. The percentage of possible minutes lost (18 h gave a potential of 1080 min of accelerometry per horse) was calculated to quantify data loss or absence.

A brief questionnaire with Likert scale responses was designed to evaluate owner acceptance of attaching the accelerometer for a period of 7 days to six possible sites, namely; the poll, withers, sternum, foreleg (above the knee), hind leg (proximal to the metatarsophalangeal joint) and sacrum. Owners were asked to indicate if they would be very willing, willing, indifferent, unwilling or very unwilling to allow attachment at each site.

### Validation study

A random selection of 25 horses wore two accelerometers located at the poll and withers while performing four different intensities of controlled PA for specified duration. It was ensured that the accelerometers did not slip, and therefore the direction of the axis most closely aligned with the vertical (gravity) did not change, by checking video footage which was synchronised to accelerometry output at each of the PA levels by setting the accelerometer and video camera to the same personal computer clock at a resolution of ±1 s. This also allowed activity levels to be confirmed retrospectively when necessary. The PA levels were selected by consideration of previous validation studies conducted in dogs and children [2, 23] but thought to be suitable for the horse;

1. *Sedentary*–when the horse was at rest in its stable, with free movement of the head and slight movement of the trunk.
2. *Light intensity physical activity*–walking the horse (four-beat gait), with slow translocation of the trunk.
3. *Moderate intensity physical activity*–a slow to fast trot (two-beat gait), with moderate translocation of the trunk.
4. *Vigorous intensity physical activity*–cantering (three-beat gait), with fast translocation of the trunk.

Sedentary behaviour was measured when horses were rested in their stable without human contact (hay and water was provided ad libitum). Light intensity PA was measured by walking horses in-hand in a sand arena at constant pace. Moderate and vigorous intensities of PA were measured as horses were lunged in a sand arena by an experienced person at constant pace. After successful completion of each stage horses were rested for at least 5 min before moving on to the next stage. The raw 15-s epochs from the accelerometer were extracted for 10 clean minutes (i.e. when a horse performed a single intensity of PA) at activity levels 1 to 3 and for 5 clean minutes at activity level 4 for each horse and then summarised as a count per minute for the vertical and integrated axis.

Accelerometry output was highly skewed, with a large number of ‘zeros’ in the sedentary behaviour category (generally while the horses stood in the stable), and so a non-parametric approach to analysis was taken in the validation study. Differences in median accelerometry output between the categories of PA described above (1–4) were tested for statistical significance using the Friedman Test, with significance level at 5 % in all tests. Where significant differences were observed with the Friedman test, the location of significant differences was tested using Wilcoxon tests between pairs of PA categories 1–4. All analyses in the validation study were conducted using commercially available software.^5^

### Categorisation of intensity of PA

Using the same sample as in the validation study, receiver operating characteristic curve (ROC) analyses were conducted using commercially available software^6^ to establish accelerometer cut points to categorise activity by intensity (sedentary, light PA, moderate PA and vigorous PA as described above). The analysis looked at each individual cpm in the data set to establish readings with the highest average sensitivity (probability of categorising a horse’s activity level correctly e.g. periods of time when the horse is actually sedentary being recorded as sedentary by the accelerometer) and specificity (probability of incorrectly categorising the activity level, e.g. light intensity PA might be recorded as sedentary). The average accelerometer cpm (both vertical and integrated axes) for each clean minute of activity recorded for all horses was modelled as the independent variable. The dependent variable was calculated by coding PA categories as either 0 or 1 depending on the boundary being generated; i.e. for sedentary behaviour this corresponded to all activities in category 1 being coded as 1 and all non-sedentary activities (categories 2–4) being coded as 0; for moderate PA this corresponded to all activities in category 3 being coded as 1 and all non-trotting activities (categories 1, 2 and 4) being coded as 0; for vigorous PA this corresponded to all activities in category 4 being coded as 1 and all non-cantering activities (categories 1–3) being coded as 0. The sedentary and moderate PA cut points provided the boundaries for the light PA category.

Contingency tables were produced and weighted Kappa (κ) calculated to determine the classification accuracy of the cut points developed in ROC analyses. κ is a measure of inter-rater agreement after agreement due to chance is taken out of the equation [24], with strength of score rated as; ‘poor’ (<0.20); ‘fair’ (0.21–0.40); ‘moderate’ (0.41–0.60); ‘good’ (0.61–0.8); and ‘very good’ (0.81–1.00) [25].

The study was approved by the University of Glasgow, School of Veterinary Medicine Ethics and Welfare Committee. Informed written consent to participation was received from each horse owner.

## Results

### Practical utility study

The convenience sample of five horses recruited to the practical utility study had a mean age of 16.8 years and height range of 1.30 to 1.65 m. Four mare’s and one gelding of varying breeds were included. Attachment of the accelerometers caused no alterations of normal behaviour although some horses proceeded to roll shortly after attachment, but quickly became accustomed to the device. Accelerometers located on the poll showed cpm during sedentary behaviour to be greater than acceleration counts recorded at other locations.

Complete 1080 min data sets were obtained from accelerometers attached to both the withers and poll. However, there were incomplete data sets for accelerometers attached at the foreleg, sternum and sacrum due to their detachment during vigorous PA.

Forty-two owners completed an owner acceptance questionnaire. The sites with the highest proportion of owners willing or very willing to allow an accelerometer to be attached were: poll (29/42 (69 %)); foreleg (27/42 (64 %)); and withers (25/42 (60 %)). The sites with the highest proportion of owners unwilling or very unwilling to allow an accelerometer to be attached were: sternum (20/42 (48 %)); sacrum (17/42 (40 %)); and hind leg (16/42 (38 %)).

In view of the findings of the practical utility assessment and owner questionnaire, the poll and withers were thought to be the most suitable sites for accelerometer attachment for the validity study.

### Validation study

**Fig. 1:**
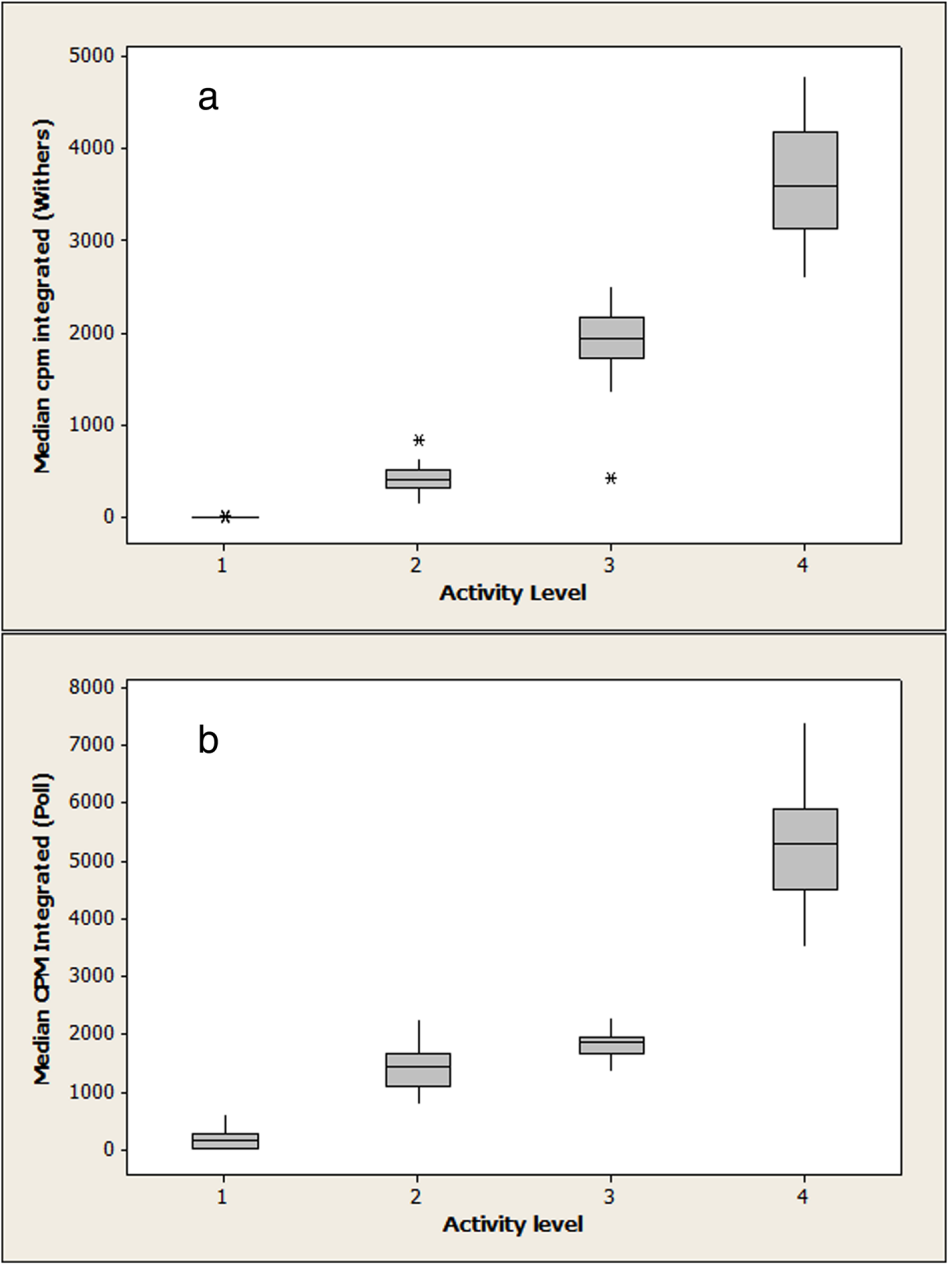
Validation study. Median count per minute from the accelerometer integrated axis during three 10-min and one 5-min periods of physical activity (PA): *1*, sedentary; *2*, light PA; *3*, moderate PA; *4*, vigorous PA for placement on the withers (**a**) and poll (**b**)

A total of 25 horses were recruited to the validation study, ranging in height from 1.57 to 1.78 m. Of the 25, five were excluded as these horses were unable to sustain clean 1 min blocks of canter. Of the remaining 20, seven were mares and 13 were geldings of varying breed and the mean age was 9.85 years. Since the accelerometry data had a non-normal distribution, non-parametric tests were used to analyse the data. Accelerometry output from the vertical axis (*P* < 0.001; data not shown) and integrated axis (*P* < 0.001) differed significantly across the four PA levels for both withers and poll placement. Medians (quartiles and range) of integrated axes accelerometry output is shown in Fig. 1 for withers and poll placement.

### Categorisation of intensity of PA; establishment of cut point determination

The results of the ROC analyses using the integrated axes output are displayed in Table 1. Using the withers placement the ROC analyses found optimal sensitivity and specificity at a cut-off of <47 cpm for sedentary behaviour, 967–2424 cpm for moderate PA and ≥2425 cpm for vigorous PA. The accuracy of the output from the withers placement in distinguishing between activity levels was very high. For the withers sedentary cut point sensitivity was 99.5 %, specificity was 100.0 % and the AUC for the ROC curve was 0.99. For the moderate PA cut point sensitivity was 96.7 %, specificity was 100.0 % and the AUC was 0.99. For the vigorous PA cut point sensitivity was 96.0 %, specificity was 97.0 % and the AUC was 0.99. Using the poll placement optimal sensitivity and specificity were found at cut-offs of <707, 1546–2609, and ≥2610 cpm for sedentary, moderate and vigorous PA respectively. Again, the accuracy of the output from the poll placement in distinguishing between PA intensities was high. For the poll sedentary cut point sensitivity was 97.5 %, specificity was 99.6 % and AUC was 0.99. For the moderate PA cut point sensitivity was 90.33 %, specificity was 79.25 % and AUC was 0.92. For the vigorous PA cut point sensitivity was 100.0 %, specificity was 100.0 % and AUC was 1.00.

**Table 1:** Sensitivity, specificity and area under the ROC curve for the development of accelerometer cut points for wither and poll placement

Placement	Activity level	Sensitivity (%)	Specificity (%)	Area under ROC curve (95 % CI)	Cut points (cpm)
Withers	Sedentary	99.5	100.0	0.999 (0.995–1.000)	0–47
	Light PA	–	–	–	48–966
	Moderate PA	96.7	100.0	0.993 (0.983–0.998)	967–2424
	Vigorous PA	96.0	97.0	0.993 (0.983–0.998)	≥2425
Poll	Sedentary	97.5	99.6	0.999 (0.993–1.000)	0–707
	Light PA	–	–	–	708–1545
	Moderate PA	90.33	79.25	0.922 (0.900–0.941)	1546–2609
	Vigorous PA	100	100	1.000 (0.995–1.000)	≥2610

(*PA* physical activity, *n* = 20)

When these cut-points were applied to the validation study data overall agreement between direct observation and accelerometer cpm was ‘very good’ as defined by κ scores [25], for both the withers (κ = 0.95, Table 2) and poll placement (κ = 0.85, Table 3). When the data using the withers placement were analysed, 197/200 (98 %) minutes were correctly classified as sedentary behaviour, with the remaining 3 min (2 %) incorrectly classified as light intensity PA. In the light intensity PA category 199/200 (99 %) minutes were correctly classified, and the remaining 1 (1 %) minute was incorrectly classified as moderate intensity PA. In the moderate intensity PA category, 171/200 (85 %) minutes were correctly classified, 10/200 (5 %) were incorrectly classified as light intensity PA, and 19/200 (10 %) minutes were incorrectly classified as vigorous intensity PA. In the vigorous intensity PA category 96/100 (96 %) minutes were correctly classified, and the remaining 4 (4 %) minutes were classified as moderate intensity PA.

**Table 2:** Classification accuracy of accelerometer when predicting PA (withers placement; *n* = 20)

		Direct observation			
		Sedentary	Light	Moderate	Vigorous
Accelerometry	Sedentary	197	0	0	0
	Light	3	199	10	0
	Moderate	0	1	171	4
	Vigorous	0	0	19	96
	Total	200	200	200	100

Measurement of agreement: κ (95 % CI) = 0.95 (0.94–0.9)

**Table 3:** Classification accuracy of accelerometer when predicting PA (poll placement; *n* = 20)

		Direct observation			
		Sedentary	Light	Moderate	Vigorous
Accelerometry	Sedentary	194	2	0	0
	Light	6	114	29	0
	Moderate	0	83	171	0
	Vigorous	0	1	0	100
	Total	200	200	200	100

Measurement of agreement: κ (95 % CI) = 0.85 (0.82–0.88)

When the data using the poll placement were analysed, 194/200 (97 %) minutes were correctly classified as sedentary behaviour, with the remaining 6 (3 %) minutes incorrectly classified as light intensity PA. In the light intensity PA category 114/200 (57 %) minutes were correctly classified, 2/200 (1 %) minutes were incorrectly classified as sedentary behavior, 83/200 (42 %) minutes were incorrectly classified as moderate intensity PA, and 1/200 (1 %) minutes was incorrectly classified as vigorous intensity PA. In the moderate intensity PA category 171/200 (85 %) minutes were correctly classified, and 29/200 (15 %) minutes were incorrectly classified as light intensity PA. Finally, in the vigorous intensity PA category, 100/100 (100 %) minutes were correctly classified. Overall 663/700 (95 %) minutes were correctly classified using the withers placement data, and 579/700 (83 %) minutes correctly classified using the poll placement data.

## Discussion

The objective assessment of PA in the horse has many useful applications in both health and disease. In this study validity of an accelerometer based activity monitor was established for sedentary, light, moderate and vigorous PA in the stabled horse and when exercised in-hand and practical utility established. The practical utility study focused on evaluating the best location to attach the accelerometer to a horse. Although all four locations of accelerometer attachment in the practical utility study were successful at recording the intensity and duration of PA, issues arose with maintaining attachment of the devices at the sacrum and sternum and hence data loss occurred. The most acceptable attachment sites were the poll and withers, using a head collar and a surcingle or nylon bib respectively. These locations and the method of attachment were well tolerated by horses and were acceptable to most owners.

While both the poll and withers could be used as sites for attachment of accelerometers for future analysis of equine PA, the withers may be preferred if only a single site of attachment is used. When measuring habitual PA in humans, accelerometers are commonly worn on the trunk, usually at the hip, as this site has been shown to provide a better measure of PA than accelerometers attached to the limbs [26]. Preliminary studies in the dog also evaluated several sites of location for accelerometers and attachment to the collar was considered most convenient and reliable [1] and is now routinely used [2–4]. Withers placement in the horse is most likely to measure PA in terms of distance travelled and movement, whilst an accelerometer attached to the poll may also measure motion not associated with traveling such as movement of the head whilst eating. This effect of ‘motion without travel’ is one reason suggested for not placing accelerometers on dogs’ limbs when assessing PA [1] and the same argument could be extrapolated to the horse. Furthermore, the better classification accuracy of the accelerometer attached to the withers (95 % of minutes classified correctly) compared to the poll attachment (83 % of minutes classified correctly) suggests that the withers may be the preferred site of attachment. Accelerometers located on the poll also showed cpm during sedentary behaviour to be greater than acceleration counts recorded at other locations which was thought to reflect movement of the head. However, it is possible that future studies may incorporate attachment at both sites during data collection. This may facilitate discrimination between whole body movements and movement of the head/neck during standing/resting.

In the absence of previous data, the optimal sample size for the validation study was difficult to assess. In human accelerometry validation studies, a sample size of 20 to 30 is typical [9–11]: analysis of a sample of 20 horses was therefore considered adequate for this study. A diverse sample of horses within a height range of 1.57 to 1.78 m was recruited to provide adequate tests of validity and to enhance the applicability of study findings. Further studies would be warranted to validate the use of accelerometry in specific equine types and breed groups should specific information be required about PA in one of these; for instance, cut points for varying PA levels may need to be determined by breed as it is likely, for example, that the threshold for vigorous exercise may be higher for a racing thoroughbred than for a pony. Other differences may also exist for the other categories of PA.

During the validation study there was excellent correlation between vertical and integrated axes suggesting that in future studies one or other of these measurements could be used, particularly if the accelerometer is attached in such a way that slippage becomes unlikely and plane of movement remains unchanged.

In order to measure both total volume and the intensity of PA among horses it is necessary to determine accelerometer cut points that could classify PA by intensity. The ability to categorise PA by intensity may in the future facilitate a clearer understanding of what particular dimensions of PA are most important for equine health. Applications may include assessment of PA in laminitis, obese individuals and geriatrics, or in post-operative horses as an indicator of pain. Due to its quantitative nature, accelerometry could also facilitate accurate assessment of the effects of clinical interventions on cumulative PA. The addition of the accelerometer used in the present study as a tool for evaluating PA also offers greater choice to the equine PA researcher interested in assessing both the total volume and intensity of PA.

There were a number of strengths and weaknesses to this study. The sample size was acceptable for the validation study, and in line with human and canine studies. Another strength was that the study investigated two important aspects of accelerometer use, namely, validation, and practical utility, both of which are important for future use of the accelerometer in equine studies. Finally the study was structured to include a range of activities that should mimic PA when horses are exercised in-hand.

A limitation could be considered to be the smaller sample size for the practical utility study and the short duration of measurement (18 h). Furthermore, the study design did not allow for the assessment of typical horse behavior when turned out in the field. Turned out horses will typically spend much of their time grazing and only move a few steps at a time before stopping again [27]. In addition, although the results presented here show that triaxial accelerometry is capable of classifying horse PA when exercised in-hand it is unclear whether it can discriminate between resting activities such as grazing and true “quiet standing” or sleep. Further studies which aim to assess the validity of accelerometry when discriminating between quiet standing and grazing, as opposed to discriminating between continuous bouts of PA of varying intensity as in the present study, are required. Another limitation of the study is that the position of the accelerometers were monitored throughout data collection which limits the applicability of the findings to studies where this is not possible. Substantial slippage of the device may result in acceleration being recorded at an unintended site and therefore invalidate the results.

It was disappointing when accelerometers became detached during the practical utility study, and this was due to activities such as rolling of the horse. It is important that this limitation of accelerometer use in the horse is recognised as, at the time of designing the study the accelerometer used cost $250 each and are therefore expensive to replace if lost or damaged. However, attention to secure attachment of accelerometers using multiple cable ties, and protection of accelerometers in fabric pockets should be considered to minimise such losses and breakages. During the validation study horses were lunged when performing activities. It is therefore possible that the resulting centripetal accelerations could be measured by the accelerometer in addition to the gravitational acceleration. However, the use of band pass filtered data may remove the centripetal acceleration. Future studies should determine whether this is the case.

The results of this study show that triaxial accelerometry is a practical and valid method for measuring PA of the horse when exercised in-hand and paves the way for future applications of triaxial accelerometry in equine research.

Further studies are required to determine whether triaxial accelerometry is valid when measuring PA in the turned out horse which would facilitate a number of studies in which habitual PA levels are of interest such as in obesity where accelerometry has been used to improve the knowledge of its aetiology, prevention and treatment [12–17, 28].

## Conclusion

This study suggests that a triaxial accelerometer is a valid device for the objective measurement of in-hand PA in horses. This technique should facilitate future research where quantitative information regarding controlled equine PA is required, and may therefore be of significant clinical and academic value.

## Footnotes

1 Actiwatch, Cambridge Neurotechnology Ltd, UK

2 Activity Lying Temperature Pedometer, Ingenieurbuero Holz, Falkenhagen, Germany

3 ActiGraph GT3X+ accelerometer, ActiGraph, Florida, USA

4 Actilife, ActiGraph, Florida, USA

5 Minitab 16.1.1, Minitab Inc. State College, PA.

6 Medcalc 12.2.1.0, Medcalc Software, Ostend, Belgium.
